# Is Mesothelioma Unrelated to the Lung Asbestos Burden? Comment on Visonà et al. Inorganic Fiber Lung Burden in Subjects with Occupational and/or Anthropogenic Environmental Asbestos Exposure in Broni (Pavia, Northern Italy): An SEM-EDS Study on Autoptic Samples. *Int. J. Environ. Res. Public Health* 2021, *18*, 2053

**DOI:** 10.3390/ijerph18137177

**Published:** 2021-07-05

**Authors:** Dario Mirabelli, Alessia Angelini, Pietro Gino Barbieri, Roberto Calisti, Fabio Capacci, Paolo Girardi, Stefano Silvestri, Anna Benedetta Somigliana

**Affiliations:** 1 Epidemiologia dei Tumori, Dipartimento di Scienze Mediche, Università di Torino e CPO-Piemonte, 10126 Torino, Italy; 2 Dipartimento di Medicina Traslazionale, Università del Piemonte Orientale, 28100 Novara, Italy; alessia.angelini72@gmail.com (A.A.); stefano.silvestri.51@gmail.com (S.S.); 3 Registro Mesoteliomi della Provincia di Brescia, Servizio di Medicina del Lavoro, Azienda Sanitaria Locale di Brescia, 25100 Brescia, Italy; pietrogino.barbieri@gmail.com; 4 SNOP Società Nazionale Operatori Prevenzione, 65122 Pescara, Italy; Roberto.Calisti@sanita.marche.it; 5 Epidemiologia Occupazionale, Servizio Salute e Sicurezza del Lavoro, Azienda Sanitaria Unica Regionale delle Marche, 62012 Civitanova Marche, Italy; 6 Prevenzione Igiene e Sicurezza dei Luoghi di Lavoro, Dipartimento di Prevenzione, Unità Sanitaria Locale Firenze, 50100 Firenze, Italy; fabio.capacci@uslcentro.toscana.it; 7 Dipartimento di Psicologia dello Sviluppo e della Socializzazione, Università di Padova, 35121 Padova, Italy; paolo.girardi@unipd.it; 8 Centro Regionale Microscopia Elettronica, Laboratorio Area Ovest–Settore Laboratori, Agenzia Regionale per la Protezione Ambientale della Lombardia, 20129 Milano, Italy; a.somigliana@arpalombardia.it

**Keywords:** asbestos, malignant mesothelioma, lung fiber burden

We read with interest the report by Visonà and coworkers on the lung asbestos fiber burden in an autopsy series of decedents from mesothelioma (MM: 59 cases) and individuals who “suffered from asbestosis and died of its complications” (13 cases) [1]. We disagree, however, with their conclusions.

Previous studies providing background knowledge were misquoted by Visonà and colleagues. They stated that “the concentration of amphibole asbestos in MM patients showed no statistically significant differences compared to controls” in the study by Wagner et al. (1982), who found indeed much higher concentrations in the lungs of asbestos textile workers—whether dying from MM or from other causes—compared with historical population controls [2]. They mentioned Rogers et al. (1991) and Sakai et al. (1994) as finding higher asbestos concentrations in the lungs of MM cases, but these two studies also showed a clear dose–response trend in MM risk [3,4]. Visonà et al. attributed such obviously relevant results only to Gilham et al.’s report (2016) [5]. Furthermore, rather than providing “proof that environmental exposure to asbestos (neighborhood and domestic) determines cumulative doses as high as those observed in some occupational exposure circumstances”, Barbieri et al. (2012) made no such comparisons [6], whereas Magnani et al. (1998) and Barbieri et al. (2020) found higher lung fiber burdens in asbestos-cement workers compared to non-occupationally exposed cases living in Casale Monferrato and Broni, respectively [7,8]. Far from denying the relevance of non-occupational exposures for MM, we suggest a more nuanced view might be more appropriate: that environmental exposures such as those occurring in Casale Monferrato and Broni in the 1950s to 1980s may have led to life-long exposures comparable to those from some—but certainly not all—occupations. The references Visonà et al. cited did not, therefore, offer support to the thesis that only “very inconsistent conclusions about the link between the concentration of asbestos in lungs and the risk of developing MM” can be drawn.

Secondly, we wish to express our concern for their decision to count only fibers with length > 5 μm “at 2000 M using backscattered electrons”, which may have prevented the identification of fibers with diameter < 0.5 μm irrespective of their length. As a consequence of such combined limitations, a large proportion of fibers—especially chrysotile—may have gone undetected with unpredictable effects on their results. By the way, the same World Health Organization document cited by the Authors mentions the potential limitations associated with the conventional counting rules in optical microscopy for airborne asbestos in the work environment [9]. Our criticism includes also the asbestos body count procedure, with the additional remark that the techniques developed and validated by a working group convened by the Italian National Institutes of Health could have been used instead [10].

Our main point, however, is the following. Visonà and colleagues claimed (i) that “the quantity of asbestos is not decisive in determining MM”, as (ii) they found “a lower amount of asbestos in lungs of people who died of MM compared to exposed people deceased from other causes”. To infer whether asbestos exposure is relevant to MM occurrence, the Authors should have compared their MM deaths (cases) with an appropriate reference series (controls), representative of the study base. This basic tenet of epidemiology may prove difficult to strictly observe in practice, particularly in hospital-based and similarly conceived studies, but at least care should be taken that controls are unaffected by exposure-related diseases (in this study: asbestos-related diseases) [11]. What the Authors actually found is not a lower lung asbestos burden in MM compared with “other causes” of death, but with asbestosis and its complications—an uninformative finding as to the inferences they wished to draw, but an otherwise rather obvious one [12]. Furthermore, they would have better addressed the dose-dependency issue by conducting a dose–response analysis, which they were prevented from doing by the absence of a suitable control series.

Their conclusions were, therefore, not just in disagreement with the literature but unwarranted, considering the limitations of their study.

## Data Availability

Not applicable.
